# Genetic Stability and Inbreeding in a Synthetic Maize Variety Based on a Finite Model

**DOI:** 10.3390/plants14020182

**Published:** 2025-01-10

**Authors:** Juan Enrique Rodríguez-Pérez, Jaime Sahagún-Castellanos, Aureliano Peña-Lomelí, Clemente Villanueva-Verduzco, Denise Arellano-Suarez

**Affiliations:** Departamento de Fitotecnia, Instituto de Horticultura, Universidad Autónoma Chapingo, km 38.5 Carretera México-Texcoco, Chapingo 56230, Estado de México, Mexico; erodriguezx@yahoo.com.mx (J.E.R.-P.); penalomeli@gmail.com (A.P.-L.); cvillanuevav@chapingo.mx (C.V.-V.); den94arellanos@gmail.com (D.A.-S.)

**Keywords:** identity by descent, different permutations, nesting, sample size

## Abstract

A synthetic variety (SV) of maize may not become stable if the sample size representing each parental line (m) is small. This research aimed to evaluate the effect of m on the inbreeding coefficient (IC) of the SV ($FSyn_{L}$) and on the stability of its genetic constitution. An SV formed by randomly mating l unrelated lines whose inbreeding coefficient is F was considered, and a random sample was taken from the genotypic array of the progeny produced by selfing a parental line $A_{1}A_{2}\;(GA)$ This sample was visualized as a set of g groups of four plants whose genotypes are all four of the GA and e represented the number of plants that failed to form a group. The ICs of the selfings and those of the intragroup and intergroup crosses were calculated to derive the formula for $FSyn_{L}$ in terms of m,g,e,l and F. It was found that as m grows, $FSyn_{L}$ tends to (1+F)/2. With m=15, $FSyn_{L}$ is practically stabilized and the probability of no genotype loss is 0.979. Moreover, the probability of losing $A_{1}$ or $A_{2}$ is practically equal to zero from m=6 onwards. However, the probability that their frequencies remain the same decreases as m gets larger.

## 1. Introduction

Synthetic varieties of maize (*Zea mayz* L.) have been recognized as a good way of using the good per se yield and general combining ability of inbred lines to form varieties with high yield and wide adaptation [1]. These varieties are also important because they are easy to produce and they maintain large amount of seed to be used the next cycle [2]. In addition, synthetic varieties (SVs) are also developed from other crop species; for example, Ciancaleoni and Negri [3] proposed a breeding strategy for the development of genetically heterogeneous and heterozygous broccoli varieties with good adaptability to diverse environmental and management conditions. These authors, starting from a synthetic variety (SV), reported a successful application of their strategy to develop new synthetic varieties for sustainable systems.

In the case of maize, SVs have the advantage of being heterogeneous and heterozygous [4], they tend to have adaptability and stable behavior [5] and the equations of Wright [6] and Busbice [7] relate their expected performance to heterozygosity. For example, in Mexico, Andrés-Meza et al. [1] evaluated 11 maize varieties in seven environments and found that six SVs exceeded the lysine control in the kernel in each of the seven environments.

Also in Mexico, Sierra-Macías et al. [8] formed crosses between five SVs and the varieties VS-536 and V-537C. All of them were classified as stable; 10 had grain yields between 6.45 and 7.26 t·ha^−1^ and outperformed the hybrid H520.

The stability of an SV is not only shown in variables such as grain yield, but it is also expected that the genotypic array of an SV (the population resulting from the random mating of its parental unrelated inbred lines [9]) is repeatable across locations and cycles, because the Hardy–Weinberg equilibrium is reached in one generation [10]. This means that the farmer who plants an SV can harvest its seed and sow it to obtain the same SV in the following cycle. In this way, there are considerable savings in seed cost compared to what would have to be paid if the farmer opted to buy the seed of a hybrid variety every cycle, since the genotypic structure of a hybrid is not repeatable from the first cycle to the next [4]. In this case, grain yields tend to decrease noticeably [4]. According to Márquez-Sánchez [11], the additional cost due to the use of hybrids in Mexico can be up to 20% of the investment in maize production. This context suggests that for agriculture practiced with limited resources, the use of SVs is a good alternative [2].

The International Maize and Wheat Improvement Center (CIMMYT) has been developing open-pollinated maize varieties for environments where different forms of environmental adversity may be encountered by smallholder farmers. The emphasis has been on improving grain yield and tolerance to adverse biotic and abiotic factors. These are synthetic varieties formed with lines resulting from CIMMYT’s work aimed at developing hybrid varieties [12]. Advances in yield and tolerance to adversity have been continuous. The success of synthetic varieties has also been observed in other contexts. For example, for drought conditions, SVs have been developed that yield at least 6.0 t·ha^−1^ [13]. Varieties of grains with high levels of provitamin A and stable behavior have also been developed in East Africa [14]. Considering that in Mexico, maize is the main source of energy and protein for its population, Andrés-Meza et al. [1] evaluated 16 SVs; of these, they found that the four highest yields fluctuated between 3.94 and 4.08 t·ha^−1^ and that in six SVs the grain yield is associated with better protein quality. In the phytosanitary context, De León-García de Alba [15] developed a SV of maize resistant to tar spot complex for the subtropical regions of Mexico. Of course, there are many other successful cases of SV development, but these also face problems in their development, including inbreeding depression, which is negatively correlated with grain yield.

Busbice [7] explains the relationship between the inbreeding coefficient and the magnitude of variables such as grain yield in maize using a formula. In this formula, the mean of the variable is expressed in terms that include the inbreeding coefficient (IC) of the SV in a linear relationship. The IC, however, is not always calculated with the required precision, particularly when the number of plants of each parent (m) is not large enough to fully satisfy expectations according to the Hardy–Weinberg law that requires “large” values of m and excludes migration, mutation and selection [10].

To derive a precise formula for the inbreeding coefficient of an SV, it must be considered that it is formed from l unrelated lines whose inbreeding coefficient is F and that the number of plants representing each parental line (m) is broken down into the number of groups (g), each formed by the genotypes that constitute the line’s progeny. It must also be considered that there are e plants that do not form a complete group. Ibarra-Sanchez et al. [4] derived a formula for the IC of an SV whose parents were t three-way line hybrids, and found that this IC is inversely related to m. It is very sensitive to changes in m when m is smaller than eight, after which it tends to stabilize rapidly. Alongside the changes in the IC due to random drift, changes in gene and genotype frequencies and even losses can also occur when m is small [10].

In this context, the need arose to contribute to minimizing these risks related to changes in gene and genotype frequencies. The aims of this research were: (a) to derive a formula to calculate $FSyn_{L}$ for any combination of values of F,m,l,g and e; (b) to derive formulas to calculate the probability that: (i) the sample from each parent includes all the genotypes contained in the genotypic array of its progeny, and (ii) no alleles are lost in the formation of the sample and that the allele frequencies of the parents are maintained. Fulfillment of these objectives should produce indicators of the size that should be assigned to m.

## 2. Results

According to Kempthorne [9], if in a population under random matting the frequencies of $A_{1}\;$and $A_{2}$ are ½ and ½, respectively, its genotype array is:

$$GA={\left[(½)A_{1}+(½)A_{2}\right]}^{2}=\left[{\left(½\right)}^{2}(A_{1}A_{1)}+¼{(A}_{1}A_{2})+¼{(A}_{2}A_{1})+{\left(½\right)}^{2}{(A}_{2}A_{2})\right]$$

Since the m plants representing a line were visualized as a random sample taken with replacements from the population whose elements are the genotypes representing the progeny of that line, if the line is $A_{1}A_{2}$, the genotypic array (GA) that produces its selfing, which is a random mating population when m=1, is:

(1) $$GA=\left(1/4\right)A_{1}A_{1}+(1/4)A_{1}A_{2}+(1/4)A_{2}A_{1}+(1/4)A_{2}A_{2}$$

Due to the nature of the genetic mechanism that controls the formation of a line’s progeny, the size m sample that represents it was considered as a set of g groups of four plants whose genotypes are those of the GA (Equation (1)). In anticipation that m is not a multiple of 4, the corresponding incomplete group formed by e plants (e=1, 2, 3) was considered.

Because the synthetic variety under study $\left({Syn}_{L}\right)$ was considered to be the population resulting from the random mating of ml plants, m from each of the l parental lines, this mating involves the random mating of the m plants from each parental line, whose inbreeding coefficient is F. This mating is the only source of inbreeding because the parental lines are not related.

Obviously, random mating between the m plants in a line includes both random mating of the four plants in each complete group and crosses between each of the four plants in each group, with each of the four plants in each of the remaining g−1 complete groups. Random mating among the four plants in a group only included four selfings and 4 × 3 crosses. Based on these considerations, formulas can be derived for: (i) the inbreeding coefficient of the ${Syn}_{L}$ $\left({FSyn}_{L}\right)$, (ii) the probability of the presence of the genotypes forming the GA (Equation (1)) in the sample [calculated using a formula for each of two approaches: (a) one that considers the inclusion of all four genotypes in the GA (Equation (1)), and (b) one that recognizes only three genotypes in the GA: two homozygotes ${(A}_{1}A_{1}\;and\;A_{2}A_{2})$ and one heterozygote $\left(A_{1}A_{2}\right)$, regardless of whether it is $A_{1}A_{2}\;or\;A_{2}A_{1}$ (calculations were based on the polynomial probability distribution and on the consideration of the different permutations of the frequencies of occurrence of the genotypes in the sample)]; and (iii) the probability of losing genes. This was based on the consideration that this event can happen only when the sample is formed with plants that have the same genotype, and that this is homozygous, i.e. either only $A_{1}A_{1}\;$or only $A_{2}A_{2}$.

### 2.1. Inbreeding Coefficient

The inbreeding coefficients of the progenies of the selfings and crosses produced by mating the four plants in each group are shown in Table 1. Based on these, the inbreeding coefficients of the crosses and selfings resulting from the random mating of the GA of Equation (1) were calculated (Table 2).

According to the data in Table 2, the averages of the inbreeding coefficients of the four progenies produced by selfing$\;{(F}_{⨂})$ and of the 12 crosses $\left(F_{C}\right)$ are, respectively:

(2) $$F_{⨂}=(3+F)/4$$

(3) $$F_{C}=(5+7F)/12$$

Another important input for the derivation of ${FSyn}_{L}$ is the average inbreeding coefficient of the progenies of each GA genotype (Equation (1)). This is (1+F)/2 (Table 2). Also contributing to the ${FSyn}_{L}$ are the progenies of the possible crosses of each of the four plants from one group, with each of the four plants from each of the remaining g−1 complete groups. The average inbreeding coefficient of the 16 crosses between the four plants of one group, and the four plants of each of the remaining g−1 complete groups of the same parent $\left(F_{CBG}\right),$ must also be (1+F)/2; that is:

(4) $$F_{CBG}=\frac{\left(1+F\right)}{2}$$

Regarding the contribution to inbreeding by the e(e=1,2,3) plants of each parent that did not manage to form a complete group, we should include the contribution generated by the e(e−1) crosses between them, direct and reciprocal, as well as the 8ge intergroup crosses between the e plants of the incomplete group and the 8g of the complete groups, 4ge direct crosses and their 4ge reciprocal crosses. These e plants will also contribute to the ${FSyn}_{L}$ with their e selfings. According to the above considerations regarding the average inbreeding coefficient of the progeny generated by random mating of the m representatives of a line (Equations (2)–(4)), ${FSyn}_{L}$ should be expressed as:

(5) $${FSyn}_{L}=\frac{m\left(3+F\right)/4+\left[12g+e\left(e-1\right)\right]\left(5+7F\right)/12+\left[16g\left(g-1\right)+8ge\right](1+F)/2}{m^{2}l}$$

Unlike previously derived formulas [16], Equation (5) expresses the exact value of the inbreeding coefficient of ${Syn}_{L}$ for any possible combination of m,g, e, F and l values. Table 3 shows the values of ${FSyn}_{L}$ for combinations of five values of F (0.000, 0.500, 0.750, 0.875, 1.000) with 24 values of m (1, 2, 3, …, 24) broken down into explicit values of F, m and e.

With respect to m, the largest changes in ${FSyn}_{L}$ for each value of F occur when the values of m are 1, 2, 3 and 4 (Table 3). However, these differences decrease as F becomes larger. When F=1, the values of ${FSyn}_{L}\;$no longer differ. Furthermore, as m grows, the differences between the values of ${FSyn}_{L}$ for the same value of F decrease and tend to stabilize, except when m is a multiple of 4 (4, 8, 12, 16, 20, 24). In this case, the values of ${FSyn}_{L}$ for each F value are equal because the frequencies of the genotypes representing each line are equal to each other, and to those of the GA (Equation (1)).

If m is a multiple of 4, the values of ${FSyn}_{L}$ are 0.500, 0.750, 0.875, 0.975 and 1.000 when F is equal to 0.000, 0.500, 0.750, 0.875 and 1.000, respectively. In contrast, for values of m that are between two consecutive multiples of 4 (when the values of *e* are 1, 2 and 3), the values of ${FSyn}_{L}$ are variable, but greater than those corresponding to a value of m that is a multiple of 4. However, this variability is less as m grows, and the values tend to the value that occurs when m is a multiple of 4. This occurs with greater speed as F becomes larger. This value of ${FSyn}_{L}$ is that of a population that is in Hardy–Weinberg equilibrium.

**Figure 1:**
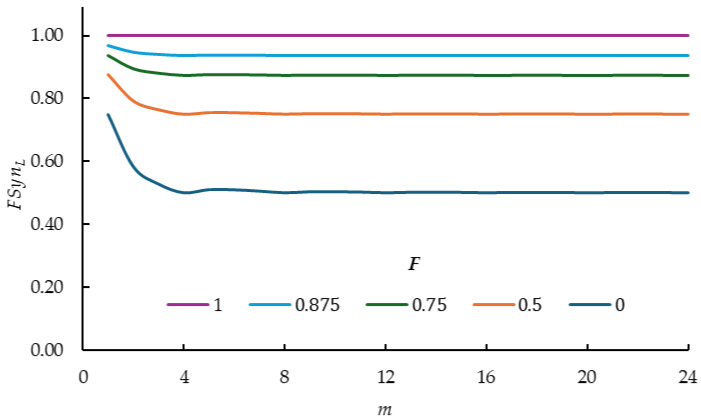
${FSyn}_{L}$ values (multiplied by 1/l) for the combinations of five inbreeding coefficients of the lines (F=0.0, 0.5, 0.75, 0.875,1.0) with 24 values of $m\left(m=1,\;2,\;3,\ldots ,\;24\right)\;$(Equation (5)).

Similar to Table 3, Figure 1 clearly shows that:

(a) For each F value the largest ${FSyn}_{L}$ values occur when m=1
(b) ${FSyn}_{L}$ increases when F is larger
(c) With m<5 ${FSyn}_{L}$ values decrease as m increases
(d) From m=5 onwards ${FSyn}_{L}$ values stabilize for each F value and their relative performance at the five F values are parallel to each other.

Relative to what happens when m<5, something similar was found by Ibarra-Sánchez et al. [4] when m<8.

### 2.2. Genotype Retention Probability

It should be noticed that if, in a set of m objects, $m_{1}$ are identical but different from the remaining objects, $m_{2}$ are identical but different from the remaining objects, …, $m_{a}$ are identical but different from the remaining objects, and $m=\sum_{i=1}^{a}m_{i}$ then the total number of different permutations is:

$$\frac{m!}{m_{1}!\;m_{2}!\ldots ..\;m_{a}!}$$

For example, if a=3:

(a) For the objects A, B and C, there are $3!/\left[\left(1!)(1!)(1!\right)\right]=6$ different permutations: ABC, ACB, BAC, BCA, CAB and CBA.
(b) For the objects A, B, A, there are $3!/[\left(2!\right)\left(1!\right)]=3$ different permutations: AAB, ABA, and BAA.

To derive a formula to calculate the probability that a sample of size m includes all four GA genotypes (Equation (1)), it must be considered that from the genotypic frequencies of the *k*-th possible sample $\left(k=1,2,\ldots ,f_{\left(m\right)}\right),$ the number of different permutations ${[}_{4,k}(NDP{)}_{m}]$ must be determined. This can be calculated with the formula:

(6) ( 4,kNDP)m=m!(k,mP1!)(k,mP2!)(k,mP3!)(k,mP4!)        ∑r=14Pr=mk,mk=1,2,…,fmPrk,m≥1

In Equation (6)  k,mPr is the number of times that the *r*-th smallest genotypic frequency (r=1, 2, 3, 4) occurs in the *k*-th set of frequencies of the genotypes that make up the size m sample. In addition, the probability that the sample includes at least one each of the four genotypes that make up the GA of Equation (1) ${(}_{4,k}P_{m})$ must be calculated for each of these frequency sets. For the *k*-th set of frequencies of this type:

(7) Pm4,k=m!(1/4)m(k,mf1!)(k,mf2!)(k,mf3!)(k,mf4!)∑q=14fqk,m =mfqk,m ≥1

In Equation (7),  k,mfq is the frequency of occurrence of genotype q;q=1, 2, 3, 4 if the genotype is $A_{1}A_{1},\;A_{1}A_{2},\;A_{2}A_{1}\;and$ $A_{2}A_{2}$, respectively.

According to Equations (6) and (7), the probability that a size m sample includes at least one of each of the four GA genotypes (Equation (1)) with the frequencies of the *k*-th frequency set $\left[P_{k}(Inclusion\;GA4{)}_{m}\right]$ is:

(8) $$P_{k}(Inclusion\;GA{4)}_{m}={[}_{4,k}(NDP{)}_{m}]{[}_{4,k}P_{m}]\;\;\;\;\;\;\;\;\;\;\;\;\;\;\;\;\;\;\;k=1,2,\ldots ,\;f_{\left(m\right)}\;\;\;\;\;\;\;\;\;\;\;\;\;\;$$

Finally, the probability that the size m sample includes all four genotypes $\left[P(Inclusion\;GA4{)}_{m}\right]$ according to Equation (8) must be calculated as:

(9) $$P(Inclusion\;GA4{)}_{m}=\sum_{k=1}^{f_{\left(m\right)}}{[}_{4,k}(NDP{)}_{m}]{[}_{4,k,}P_{m}]$$

Using Equation (9), the probability that a size 15 sample includes at least one each one of the four GA genotypes (Equation (1)) was calculated to be 0.9467. Inclusion of all four GA genotypes (Equation (1)) in the sample certainly ensures that no loss of genetic material occurs. However, since $A_{1}A_{2}$ and $A_{2}A_{1}$ are genetically equal, if both are represented only as $A_{1}A_{2}$, the GA (Equation (1)) can alternatively be represented as GA3 in the form:

(10) $$GA3=(1⁄4)\;A_{1}A_{1}+(1⁄2)A_{1}A_{2}+(1⁄4)\;A_{2}A_{2}$$

It is clear that a random sample can contain all three genotypes of GA3 (Equation (10)) from m=3. With this sample size, the probability that these three genotypes are included in the sample is 0.1875. With larger samples the probability should be higher. In general, the methodology used to derive a formula for calculating the probability that the sample contains all three GA3 genotypes is similar to that used to derive the probability that the sample includes all four GA genotypes (Equation (1)), which is concluded in Equation (9). When m=6, for example, genotypes $A_{1}A_{1}$, $A_{1}A_{2}$ and $A_{2}A_{2}$ can be included with the frequencies of any one of the following three different sets of frequencies: (1)$\;\left\{1,\;2,\;3\right\}$, (2) $\left\{1,\;1\;,4\right\}$ and (3) $\left\{2,\;2,\;2\right\}$. In each of these the order of the numbers refers to the frequency of $A_{1}A_{1}$, $A_{1}A_{2}$ and $A_{2}A_{2}$, respectively. In each set, different permutations of the genotypic frequencies must be considered. For example, in the first case $\left[(1)\;\left\{1,\;2,\;3\right\}\right]$ there are six different permutations of its frequencies: $\left(1)\;\left\{1,\;2,\;3\right\};(2)\;\left\{1,\;3,\;2\right\};(3)\;\left\{2,\;1,\;3\right\};(4)\;\left\{2,\;3,\;1\right\};(5)\;\left\{3,\;1,\;2\right\}\;and\;(6)\;\{3,\;2,\;1\right\}$. In each of these six different permutations, here and hereafter, the numbers constituting each permutation are the frequencies, in the same order, of $A_{1}A_{1}$, $A_{1}A_{2}$ and $A_{2}A_{2}$ (Equation (10)). The different permutations of the frequency set $(2)\;\left\{1,\;1,\;4\right\}$ are: $\left(1)\left\{1,1,4\right\},(2)\{1,4,1\},\;and\;(3)\{4,1,1\right\}$. On the other hand, the set (3) {4,4,4} has only one “permutation”: {2,2,2}. To calculate the probability that the sample includes genotypes $A_{1}A_{1}$, $A_{1}A_{2}$ and $A_{2}A_{2}$, it was considered that the number of different sets of genotypic frequencies depends on $m\;\left[{f\prime }_{\left(m\right)}\right]$ and that the number of different permutations of the case $k\;{[}_{3,k}(NDP{)}_{m}]$ can be calculated according to the formula:

(11) (3,kNDP)m=m!(k,mP1!)(k,mP2!)(k,mP3!)∑t=13Pt=mk,mk=1, 2, 3, …,f(m)′Ptk,m≥1

where  k,mPt is the number of times the *t*-th smallest genotypic frequency $\left(t=1,\;2,\;3\right)$ occurs.

Evidently, the different permutations of the *k-*frequency set are nested in that set, and if i(k) is the *t*-th different permutation of the *k*-th set, the number of different permutations in this case is a function of $k\;\left[g_{\left(k\right)}\right]$; that is, $\left(i\right)k=1,\;2,\;\ldots ,\;g_{\left(k\right)}.$

If $P_{\left(i\right)k}$ represents the probability that the *i*-th different permutation of the *k*-th set of genotypic frequencies in the sample includes at least one of each of the three genotypes in a size m sample, and if in that sample $f_{\left(i\right)k1},f_{\left(i\right)k2}$ and $f_{\left(i\right)k3}$ are the frequencies of genotypes $A_{1}A_{1}$, $A_{1}A_{2}$ and $A_{2}A_{2}$, respectively, then:

(12) $$\begin{array}{cc} P_{\left(i\right)k}=\frac{m!(¼{)}^{f_{\left(i\right)k1}+f_{\left(i\right)k3}}(½{)}^{f_{\left(i\right)k2}}}{\left(f_{\left(i\right)k1}!)(f_{\left(i\right)k2}!)(f_{\left(i\right)k3}!\right)} & k=1,\;2,\ldots ,{f\prime }_{\left(m\right)}\\ & \sum_{j=1}^{3}f_{\left(i\right)kj}=m\\ & f_{{{\left(i\right)}_{k}}_{j}\;\geq \;1} \end{array}$$

A formula for the probability that the size *m* sample includes at least one of each of the 3 GA3 genotypes will now be derived $\left[P(Inclusion\;GA3{)}_{m}\right]$. Since $P_{\left(i\right)k}$ is the probability of such an event occurring in the *i*-th different permutation of the *k*-th frequency set, then as $k=1,\;2,\;\ldots ,\;{f\prime }_{\left(m\right)}$, by Equation (12):

(13) $$P\left[(Inclusion\;GA3{)}_{m}\right]=\sum_{k=1}^{f_{\left(m\right)}^{\prime }}\sum_{\left(i\right)k}^{g_{\left(k\right)}}\frac{m!(¼{)}^{f_{\left(i\right)k1}+f_{\left(i\right)k3}}({½)}^{f_{\left(i\right)k2}}}{\left(f_{\left(i\right)k1}!)(f_{\left(i\right)k2}!)(f_{\left(i\right)k3}!\right)}\;$$

Or, more briefly, based on Equation (12):

(14) $$P\left[(Inclusion\;GA3{)}_{m}\right]=\sum_{k=1}^{f_{\left(m\right)}^{\prime }}\sum_{\left(i\right)k=1}^{g_{\left(k\right)}}P_{\left(i\right)k}\;$$

Table 4 shows the calculation of the probabilities that are part of the probability that a size 6 sample (m=6) contains at least one of each of the genotypes $A_{1}A_{1}$, $A_{1}A_{2}$ and $A_{2}A_{2}$. Note that when m=6, the probability of including the three genotypes (0.646) can hardly be considered satisfactory for practical purposes.

When m=9, the probability rises to 0.845 (Equation (12)). When m=15 the probability is already considerable, 0.979 (Table 5). It should be considered, however, that even with sample sizes such as m=12 and m=15, the samples can be formed with genotypes whose frequencies of $A_{1}\;and\;A_{2}$ are very different and have an increased inbreeding coefficient. For example, in the two extreme cases of genotypic frequencies: (a) 1, 1, 10 and (b) 10, 1, 1 both for $A_{1}A_{1}$, $A_{1}A_{2}$ and $A_{2}A_{2}$, respectively, the frequencies of $A_{1}$, in (a) and of $A_{2}$ in (b) are very low, although the joint probability of occurrence of these two different permutations must also be very low.

For a size 12 sample, 12 different sets of frequencies of $A_{1}A_{1}$, $A_{1}A_{2}$ and $A_{2}A_{2}\;$are possible. Each set, except when the frequency of each genotype is $4\;\left\{4,\;4,\;4\right\}$, has several different permutations. There are seven sets of three different frequencies and from each of them, six different permutations are generated, while from each of the remaining four sets of different frequencies, only three different permutations are possible.

The probability that the sample includes the four genotypes $A_{1}A_{1},\;A_{1}A_{2},\;A_{2}A_{1}$ and $A_{2}A_{2}\;\left[P(Inclusion\;GA4{)}_{m}\right]$ should be lower than the probability that it includes the genotypes displayed as three, i.e., two homozygotes and one heterozygote with frequencies of ¼ and ½, respectively $\left[P(InclusionGA3{)}_{m}\right]$. This is because events that would not be included in *GA4* can be included in the second case. For example, the cases in which the frequencies in *GA3* (Equation (10)) for $A_{1}A_{1}$, $A_{1}A_{2}$ and $A_{2}A_{2}$ are a, b and c (a≠0, b≠0 and c≠0), respectively, in GA4 they can be, among others things, in the form a, b, 0, c, and a, 0, b, c, for $A_{1}A_{1}$, $A_{1}A_{2},\;A_{2}A_{1}\;and\;A_{2}A_{2}$, respectively. Because of such cases, $P(Inclusion\;GA3{)}_{m}>P(Inclusion\;GA4{)}_{m}$. Obviously in both cases there is no gene loss and the frequencies of $A_{1}$ and $A_{2}$ are equal.

### 2.3. Probability of No Exclusion of Genes from the Sample

There may be samples in which m plants have the same genotype and yet there is no gene loss. Such a case occurs when that genotype is $A_{1}A_{2}$. However, a gene would be lost if the m plants in the sample had only genotype $A_{1}A_{1}$ or only $A_{2}A_{2}$. This can happen with a probability of $2(¼{)}^{m}$, regardless of the genotypic array under consideration (Equations (1) and (10)). Only in these two cases can gene loss occur. However, the probability of this occurring is practically negligible, from $m=5:\;2{\left(0.25\right)}^{5}=0.0019$.

It should be evident that the probability of losing a gene from each parent is also the probability that the inbreeding coefficient of the sample reaches its maximum value (100%).

## 3. Discussion

It is striking that the smallest ${FSyn}_{L}$ value occurs whenever *m* is a multiple of 4 (Table 3). Of course, it should not be interpreted that a size 4 sample is sufficient to represent a parental line of the ${Syn}_{L}$ just because its ${FSyn}_{L}$ is the smallest. With a random size 4 sample, it is most likely that all four plants will have the same homozygous genotype, that is, only $A_{1}A_{1}$ or only $A_{2}A_{2}$. In the first and second cases, $A_{2}$ and $A_{1\;}$would be lost, respectively. In either case there would be an increase in the inbreeding coefficient. Moreover, to avoid this, it seems logical to use a larger sample size, but ideally one that does not exceed a larger size than necessary.

It does not seem appropriate, or possible, to determine only the exact minimum value of m that is compatible with the concept of a “large sample”. However, it is possible to determine a value of m from which the value of ${FSyn}_{L}$ is reasonably considered acceptable, with a probability that satisfies the breeder’s requirements. The changes in ${FSyn}_{L}$ are already very small from m=8 (Table 3). For this value of m, or one close to it, it should be useful to know the probability that all GA genotypes are retained in the sample. This ensures that no loss of genetic material occurs due to random sampling and that, consequently, the inbreeding coefficient does not increase. It may seem reasonable to think that samples with more uniform genotypic frequencies tend to contribute more to the probability that no genotype is lost. However, it should also be considered that with more uniform frequencies, the different permutations tend to be less numerous and thus their contribution to the probability is not large. For example, for m=15 the set of genotypic frequencies $\left\{\left.3,\;4,\;4,\;4\right\}\right.$ has only four different permutations, and their joint contribution to the probability that no genotypes are lost is 0.0587 (Equation (8)). In contrast, with frequencies $\left\{\left.3,\;3,\;4,\;5\right\}\right.$ the probability rises to 0.140956.

When instead of the GA (Equation (1)) one considers the genotypic array GA3 (Equation (10)) formed by the genotypes ${\;A}_{1}A_{1},\;A_{1}A_{2}\;and\;A_{2}A_{2}\;$with probabilities ¼, ½ and ¼, respectively, the samples most likely to include the three genotypes are those formed by more similar genotypic frequencies but with small differences, as is the case when the four genotypes of Equation (1) were considered. For example, Table 5, for m=15, shows that with the group of genotypic frequencies in the sample consisting of the numbers 4, 5 and 6, including the six different permutations, the probability that it includes the three genotypes is 0.1316. In contrast, with frequencies 1, 1, 13, with their three different permutations, the probability is only 0.0016.

Consider sample size 15 consisting of genotypes $A_{1}A_{1},\;A_{1}A_{2},\;A_{2}A_{1}$ and $A_{2}A_{2}$ with frequencies 1,1,12 and 1 (GA, Equation(1)), respectively. In this case the frequencies of $A_{1}\;and\;A_{2}$ are both 1/2. Therefore, the progeny resulting from random mating of the 15 plants that make up this sample should have the following genotypic array: $(1/4)\;A_{1}A_{1}+(1/4)A_{1}A_{2}+(1/4)A_{2}A_{1}+(1/4)A_{2}A_{2}$. The inbreeding coefficient of this progeny is 0.5+0.5F (Table 1). If, instead, the sample frequencies of $A_{1}A_{1},\;A_{1}A_{2},\;A_{2}A_{1}\;and\;A_{2}A_{2}$ were 1, 1, 1 and 12, respectively, the frequencies of $A_{1}\;and\;A_{2}$ would be 2/15 and 13/15, respectively. With these, the progeny produced by the random mating of the plants forming the sample would have the following expected genotypic array: $(4/225)\;A_{1}A_{1}+(26/225)A_{1}A_{2}+(26/225)A_{2}A_{1}+(169/225)A_{2}A_{2}$. The inbreeding coefficient of this progeny is 4/225+(26/225)F+(26/225)F+169/225=0.77+0.23 F. The considerable difference between these two inbreeding coefficients is evidence of the strong impact that different permutations of a set of genotypic frequencies can have. These two cases of different permutations, however, occur with the same probability during sample formation $\left[0.0000025\;\left(Equation(9)\right)\right]$. If, instead, the frequencies were not so different, large inbreeding coefficients would not be expected.

A desirable quality of a sample of m representatives of each parent in this particular case is the equality of frequencies of $A_{1}$ and of $A_{2}$. With this, the expected genotypic array produced by random mating of the l parents is that of the intended synthetic variety $\left(Syn_{L}\right)$. Regarding a parent, as a result of random sampling of size m, the frequencies of $A_{1}$ and $A_{2}$ may or may not be equal. When they are equal, the contribution of that parent to $FSyn_{L}$ is minimized.

In a size 2 sample, for the frequencies of $A_{1}$ and $A_{2}$ in the random sample to be equal, the sample must consist of either (a) two plants of genotype $A_{1}A_{2}$ or (b) one $A_{1}A_{1}$ and the other $A_{2}A_{2}$. The probability of either of these events occurring is (½)(½)+2(¼)(¼)=0.375. For m=3, m=4 and m=5, the probabilities of occurrence of equal gene frequencies are 0.3125, 0.2968 and 0.2460, respectively. For m=20 the probability is considerably reduced (0.125).

The above results in the context of the desirability of the frequencies of $A_{1}$ and $A_{2}$ being equal show a clear trend: as *m* increases the probability of this happening decreases. It gets increasingly closer to what seems to be its destiny: zero.

## 4. Materials and Methods

Due to the reproductive characteristics of maize, a diploid, monoecious and allogamous species, its reproductive mode was considered to fit the random mating model. Regarding the development of the synthetic variety of maize being studied, its origin was considered to be that of the population generated by the random mating of l unrelated lines whose inbreeding coefficient is F (0≤F≤1), each represented by *m* plants. Regarding *F*, the above means that if a random plant whose genotype is $A_{1}A_{2}\;$ is taken from the progeny of a line, the probability (Ρ) that genes $A_{1}\;andA_{2}$ are identical by descent $\left(\equiv \right)$ is F; that is, $Ρ\left(A_{1}\equiv A_{2}\right)=F$ [4].

Because the lines are unrelated, ${FSyn}_{L}$ was derived based on the consideration that the sources of inbreeding were only those resulting from the random mating of the m plants that represent each line (selfing and crosses). The probability of the presence of the genotypes forming the genotypic array of Equation (1) in the m size sample was calculated based on the polynomial probability distribution. Finally, the probability of losing genes was calculated based on the consideration that these events occur only when all genotypes in the sample are either only $A_{1}A_{1}$ or only $A_{2}A_{2}$.

## 5. Conclusions

A synthetic variety formed by the random mating of l unrelated lines was studied, where the inbreeding coefficient is F, and where each line is represented by m plants taken at random with replacement of the genotypes forming the genotypic array of each parental line. For the first time, a formula for the inbreeding coefficient of this synthetic variety was derived in terms of m, F, g, l and e, where g is the number of groups of four plants whose genotypes are those of the GA (Equation (1)) and e is the number of plants that did not complete a group $\left(FSyn_{L}\right)$. According to this formula, $FSyn_{L}$ takes the same value whenever m is a multiple of 4 and takes larger values different from each other for m values that are between two consecutive multiples of 4. However, as m grows, these values lose variability and tend toward the value that $FSyn_{L}$ has when m is a multiple of 4. Furthermore, according to the derived formulas, for the probability that no genotype is lost during sample formation to be 0.936 or 0.979, m must be equal to 12 and 15, respectively.

Another result of this research is that the probability that $A_{1}$ or $A_{2}$ is not lost is practically equal to 1 from m=5 onwards. Finally, it was shown that the probability that the frequencies of $A_{1}$ and $A_{2}$ in the sample are equal decreases as *m* becomes larger, with an apparently inexorable tendency toward 0.

## Figures and Tables

**Table 1:** Inbreeding coefficients of the progenies produced by random mating of the four plants whose genotypes are those of the GA (Equation (1)).

Reproductive Events ^¶^	Progenies	Inbreeding Coefficients	
$A_{1}A_{1⨂}$	$A_{1}A_{1}$	(1)(1)	=1
$A_{2}A_{2⨂}$	$A_{2}A_{2}$	(1)(1)	=1
$A_{1}A_{2⨂}$	$\left(1/4\right)A_{1}A_{1}+\left(1/2\right)A_{1}A_{2}+\left(1/4\right)A_{2}A_{2}$	$\left(1/4\right)\left(1\right)+\left(1/2\right)\left(F\right)+\left(1/4\right)\left(1\right)\;\;\;\;\;\;\;\;\;\;\;\;\;\;\;\;\;\;\;\;$	=(1+F)/2
$A_{2\;}A_{1⨂}$	$\left(1/4\right)A_{2}A_{2}+\left(1/2\right)A_{2}A_{1}+\left(1/4\right)A_{1}A_{1}$	$\left(1/4\right)\left(1\right)+\left(1/2\right)\left(F\right)+\left(1/4\right)\left(1\right)\;\;\;\;\;\;\;\;\;\;\;\;\;\;\;\;\;\;\;\;$	=(1+F)/2
$A_{1}A_{1}xA_{2}A_{2}$	$A_{1}A_{2}$	(1)(F)	=F
$A_{1}A_{1}xA_{1}A_{2}$	$\left(1/2\right)A_{1}A_{1}+\left(1/2\right)A_{1}A_{2}$	$\left(1/2\right)\left(1\right)+\left(1/2\right)\left(F\right)\;\;\;\;\;\;\;\;\;\;\;\;\;\;\;\;\;\;\;\;\;\;\;\;\;\;\;\;\;\;\;\;\;\;\;\;\;\;\;\;\;\;$	=(1+F)/2
$A_{1}A_{1}xA_{2}A_{1}$	$\left(1/2\right)A_{1}A_{2}+\left(1/2\right)A_{1}A_{1}$	$\left(1/2\right)F+\left(1/2\right)\left(1\right)\;\;\;\;\;\;\;\;\;\;\;\;\;\;\;\;\;\;\;\;\;\;\;\;\;\;\;\;\;\;\;\;\;\;\;\;\;\;\;\;\;\;\;\;\;\;$	=(1+F)/2
$A_{1}A_{2}xA_{2}A_{1}$	$\left(1/4\right)A_{1}A_{2}+\left(1/4\right)A_{1}A_{1}+\left(1/4\right)A_{2}A_{2}+\left(1/4\right)A_{2}A_{1}$	$\left(1/4\right)F+\left(1/4\right)\left(1\right)+\left(1/4\right)\left(1\right)+(1/4)(F)$	=(1+F)/2
$A_{1}A_{2}xA_{2}A_{2}$	$\left(1/2\right)A_{1}A_{2}+\left(1/2\right)A_{2}A_{2}$	$\left(1/2\right)F+\left(1/2\right)\left(1\right)\;\;\;\;\;\;\;\;\;\;\;\;\;\;\;\;\;\;\;\;\;\;\;\;\;\;\;\;\;\;\;\;\;\;\;\;\;\;\;\;\;\;\;\;\;\;$	=(1+F)/2
$A_{2}A_{1}xA_{2}A_{2}$	$\left(1/2\right)A_{2}A_{2}+\left(1/2\right)A_{1}A_{2}$	$\left(1/2\right)\left(1\right)+\left(1/2\right)F\;\;\;\;\;\;\;\;\;\;\;\;\;\;\;\;\;\;\;\;\;\;\;\;\;\;\;\;\;\;\;\;\;\;\;\;\;\;\;\;\;\;\;\;\;\;$	=(1+F)/2

^¶^ selfings (⨂) and crosses $\left(x\right)$. Reciprocal crosses are omitted.

**Table 2:** Average inbreeding coefficients of the crosses and selfings produced by the random mating of the four plants of a group represented by the genotypes that form the GA that produces a line (Equation (1)).

Genotypes					Average ^¶^
Genotypes	$A_{1}A_{1}$	$A_{1}A_{2}$	$A_{2}A_{1}$	$A_{2}A_{2}$	
$A_{1}A_{1}$	1	(1+F)/2	(1+F)/2	F	(1+F)/2
$A_{1}A_{2}$	(1+F)/2	(1+F)/2	(1+F)/2	(1+F)/2	(1+F)/2
$A_{2}A_{1}$	(1+F)/2	(1+F)/2	(1+F)/2	(1+F)/2	(1+F)/2
$A_{2}A_{2}$	F	(1+F)/2	(1+F)/2	1	(1+F)/2
Average	(1+F)/2	(1+F)/2	(1+F)/2	(1+F)/2	(1+F)/2

F¶ is the inbreeding coefficient of the line $A_{1}A_{2}.$

**Table 3:** Inbreeding coefficient of a synthetic variety formed by random mating of the m plants from each of l unrelated lines $\left({FSyn}_{L}\right)$. Results are shown for combinations of 24 values of m with five inbreeding coefficients (F) of the lines (Equation (5)) ^¶^.

F						
m	e	0.0000	0.5000	0.7500	0.8750	1.0000
1	1	0.75	0.88	0.94	0.97	1.00
2	2	0.58	0.79	0.90	0.95	1.00
3	3	0.53	0.76	0.88	0.94	1.00
4	0	0.50	0.75	0.88	0.94	1.00
5	1	0.51	0.76	0.88	0.94	1.00
6	2	0.51	0.75	0.88	0.94	1.00
7	3	0.51	0.75	0.88	0.94	1.00
8	0	0.50	0.75	0.88	0.94	1.00
9	1	0.50	0.75	0.88	0.94	1.00
10	2	0.50	0.75	0.88	0.94	1.00
11	3	0.50	0.75	0.88	0.94	1.00
12	0	0.50	0.75	0.88	0.94	1.00
13	1	0.50	0.75	0.88	0.94	1.00
14	2	0.50	0.75	0.88	0.94	1.00
15	3	0.50	0.75	0.88	0.94	1.00
16	0	0.50	0.75	0.88	0.94	1.00
17	1	0.50	0.75	0.88	0.94	1.00
18	2	0.50	0.75	0.88	0.94	1.00
19	3	0.50	0.75	0.88	0.94	1.00
20	0	0.50	0.75	0.88	0.94	1.00
21	1	0.50	0.75	0.88	0.94	1.00
22	2	0.50	0.75	0.88	0.94	1.00
23	3	0.50	0.75	0.88	0.94	1.00
24	0	0.50	0.75	0.88	0.94	1.00

^¶^ The ${FSyn}_{L}$ values are obtained by multiplying the inbreeding coefficients in the table by (1/l). In this table *e* is the number of plants that failed to form a group (e=m−4g) and g is the number of complete groups. Each group consists of the four plants whose genotypes are those of the GA (Equation (1)).

**Table 4:** Calculation of the probability that a random size 6 sample of the progeny of line $A_{1}A_{2}$ (Equation (10)) contains the genotypes $A_{1}A_{1}$, $A_{1}A_{2}$ and $A_{2}A_{2}$. The procedure was based on the calculation of the probability of inclusion of the three sets of frequencies $\left(P_{\left(i\right)k};\;Equations\;(12)-(14)\right)$.

	Genotypic Frequencies			$P_{\left(i\right)k}$		
$\left(i\right)k$	$A_{1}A_{1}$	$A_{1}A_{2}$	$A_{2}A_{2}$			
	k=1					
$\left(1\right)1$	1	2	3	$6!\left[(1/4)(1/2{)}^{2}(1/4{)}^{3}\right]/(1!2!3!)$	=	0.059
$\left(2\right)1$	1	3	2	$6!\left[(1/4)(1/2{)}^{3}(1/4{)}^{2}\right]/(1!3!2!)$	=	0.117
$\left(3\right)1$	2	1	3	$6!\left[(1/4{)}^{2}(1/2)(1/4{)}^{3}\right]/(2!1!3!)$	=	0.029
$\left(4\right)1$	2	3	1	$6!\left[\left(1/4{)}^{2}(1/2{)}^{3}(1/4\right)\right]/(2!3!1!)$	=	0.117
$\left(5\right)1$	3	1	2	$6!\left[(1/4{)}^{3}(1/2)(1/4{)}^{2}\right]/(3!1!2!)$	=	0.029
$\left(6\right)1$	3	2	1	$6!\left[\left(1/4{)}^{3}(1/2{)}^{2}(1/4\right)\right]/(3!2!1!)$	=	0.059
$\sum_{\left(i\right)1=1}^{6}P_{\left(i\right)1}$						0.411
	k=2					
$\left(1\right)2$	1	1	4	$6!\left[(1/4)(1/2)(1/4{)}^{4}\right]/(1!1!4!)$	=	0.015
$\;\left(2\right)2$	1	4	1	$6!\left[\left(1/4)(1/2{)}^{4}(1/4\right)\right]/(1!4!1!)$	=	0.117
$\left(3\right)2$	4	1	1	$6!\left[\left(1/4{)}^{4}(1/2)(1/4\right)\right]/(1!1!4!)$	=	0.015
$\sum_{\left(i\right)2=1}^{3}P_{\left(i\right)2}$						0.147
	k=3					
$\left(1\right)3$	2	2	2	$6!\left[(1/4{)}^{2}(1/2{)}^{2}(1/4{)}^{2}\right]/(2!2!2!)$	=	0.088
$\sum_{\left(i\right)3=1}^{1}P_{\left(i\right)3}$						0.088
$\sum_{k=1}^{3}\sum_{\left(i\right)k=1}^{g(k)}P_{\left(i\right)k}$						0.646

**Table 5:** Probability that a size 15 sample includes the genotypes $A_{1}A_{1}$, $A_{1}A_{2}$ and $A_{2}A_{2}$ of GA3 (Equation (10)). $P_{\left(i\right)k}$ is the probability that the *i*-th different permutation of the *k*-th set of genotypic frequencies occurs and $\sum_{\left(i\right)k=1}^{g(k)}P_{\left(i\right)k\;}$ is the sum of probabilities of the different permutations of the k-frequency set (Equations (12)–(14)).

	Genotypic Frequencies			Number of Different Permutations (Equation (11))	$\sum_{\left(i\right)k=1}^{g(k)}P_{\left(i\right)k}$		
*k*	$A_{1}A_{1}$	$A_{1}A_{2}$	$A_{2}A_{2}$				
1	5	5	5	1	$\left[15!/5!5!5!\right]\left[(0.25{)}^{10}(0.5{)}^{5}\right]$	=	0.0225
2	1	1	13	3	$\left[15!/13!\right]\left[2(0.25{)}^{14}(0.5)+2(0.25{)}^{2}(0.5{)}^{13}\right]$	=	0.0016
3	1	7	7	3	$\left[15!/7!7!)\right]\left[2(0.25{)}^{8}(0.5{)}^{7}+(0.25{)}^{14}(0.5)\right]$	=	0.0123
4	4	4	7	3	$\left[15!/7!4!4!\right]\left[2(0.25{)}^{11}(0.5{)}^{4}+(0.25{)}^{8}(0.5{)}^{7}\right]$	=	0.0671
5	2	2	11	3	$\left[15/2!2!11!\right]\left[2(0.25{)}^{13}(0.5{)}^{2}+(0.25{)}^{11}(0.5{)}^{4}\right]$	=	0.0157
6	3	6	6	3	$\left[15!/6!6!3!\right]\left[2(0.25{)}^{9}(0.5{)}^{6}+(0.25{)}^{12}(0.5{)}^{3}\right]$	=	0.0532
7	3	3	9	3	$\left[15!/3!3!9!\right]\left[2(0.25{)}^{12}(0.5{)}^{3}]+(0.25{)}^{6}(0.5{)}^{9}\right]$	=	0.0492
8	1	2	12	6	$\left[15!/2!12!\right]\left[2(0.25{)}^{13}(0.5{)}^{2}+2(0.25{)}^{14}(0.5)+2(0.25{)}^{3}(0.5{)}^{12}\right]$	=	0.0104
9	1	3	11	6	$\left[15!/11!3!\right]\left[2(0.25{)}^{12}(0.5{)}^{3}+2(0.25{)}^{14}\left(0.5\right)+2(0.25{)}^{4}(0.5{)}^{11}\right]$	=	0.0209
10	1	4	10	6	$\left[15!/4!10!\right]\left[2(0.25{)}^{11}(0.5{)}^{4}+2(0.25{)}^{14}\left(0.5\right)+2(0.25{)}^{5}(0.5{)}^{10}\right]$	=	0.0291
11	1	5	9	6	$\left[15!/5!9!\right]\left[2(0.25{)}^{10}(0.5{)}^{5}+2(0.25{)}^{14}\left(0.5\right)+2(0.25{)}^{6}(0.5{)}^{9}\right]$	=	0.0305
12	1	6	8	6	$\left[15!/6!8!\right]\left[2(0.25{)}^{9}(0.5{)}^{6}+2(0.25{)}^{14}(0.5)+2(0.25{)}^{7}(0.5{)}^{8}\right]$	=	0.0270
13	2	6	7	6	$\left[15!/2!6!7!\right]\left[2(0.25{)}^{9}(0.5{)}^{6}+2(0.25{)}^{13}(0.5{)}^{2}+2(0.25{)}^{8}(0.5{)}^{7}\right]$	=	0.0657
14	2	3	10	6	$\left[15!/2!3!10!\right]\left[2(0.25{)}^{12}(0.5{)}^{3}+2(0.25{)}^{13}(0.5{)}^{2}+2(0.25{)}^{5}(0.5{)}^{10}\right]$	=	0.0580
15	2	4	9	6	$\left[15!/2!4!9!\right]\left[2(0.25{)}^{11}(0.5{)}^{4}+2(0.25{)}^{13}(0.5{)}^{2}+2(0.25{)}^{6}(0.5{)}^{9}\right]$	=	0.0722
16	2	5	8	6	$\left[15!/2!5!8!\right]\left[2(0.25{)}^{10}(0.5{)}^{5}+2(0.25{)}^{13}(0.5{)}^{2}+2(0.25{)}^{7}(0.5{)}^{8}\right]$	=	0.0734
17	3	4	8	6	$\left[15!/3!4!8!\right]\left[2(0.25{)}^{11}(0.5{)}^{4}+2(0.25{)}^{12}(0.5{)}^{3}+2(0.25{)}^{7}(0.5{)}^{8}\right]$	=	0.1174
18	4	5	6	6	$\left[15!/4!5!6!\right]\left[2(0.25{)}^{10}(0.5{)}^{5}+2(0.25{)}^{11}(0.5{)}^{4}+2(0.25{)}^{9}(0.5{)}^{6}\right]$	=	0.1316
19	3	5	7	6	$\left[15!/3!5!7!\right]\left[2(0.25{)}^{10}(0.5{)}^{5}+2(0.25{)}^{12}(0.5{)}^{3}+2(0.25{)}^{8}(0.5{)}^{7}\right]$	=	0.1127
$\sum_{k=1}^{19}\sum_{\left(i\right)k=1}^{g(k)}P\left(i\right)k$							0.979

## Data Availability

Data is contained within the article.
